# Supplementary material for: Investigating the influence of Diadematidae scuticociliatosis on host microbiome composition
Source: mSystems. 2025 Feb 19;10(3):e01418-24. doi: 10.1128/msystems.01418-24 (PMC11915805; doi:10.1128/msystems.01418-24)
Supplement: Supplemental material — Supplemental figures and table. [file msystems.01418-24-s0001.docx]

Supplementary Material:

**Supplementary Table 1.** Characteristics of field-collected specimens from the Caribbean and Réunion Island (Western Indian Ocean, France)

| **Ocean** | **Jurisdiction** | **Species** | **Health** | **Tissue Type** |
| --- | --- | --- | --- | --- |
| **Caribbean Sea** | Antigua and Barbuda | Diadema antillarum | Normal at Affected Site | Spines (n = 10) |
|  |  |  | Abnormal | Spines (n = 5) |
|  | Grenada | Diadema antillarum | Normal at Affected Site | Body Wall (n = 3) |
|  |  |  |  | Coelomic Fluid (n = 3) |
|  |  |  |  | Gonad (n = 3) |
|  |  |  |  | Intestine (n = 3) |
|  |  |  |  | Spine (n = 3) |
|  |  |  | Abnormal | Body Wall (n = 3) |
|  |  |  |  | Coelomic Fluid (n = 2) |
|  |  |  |  | Gonad (n = 3) |
|  |  |  |  | Intestine (n = 3) |
|  |  |  |  | Spine (n = 3) |
|  | Saba, Caribbean Netherlands | Diadema antillarum | Reference | Body Wall (n = 3) |
|  |  |  |  | Coelomic Fluid (n = 1) |
|  |  |  |  | Gonad (n = 3) |
|  |  |  |  | Intestine (n = 3) |
|  |  |  | Normal at Affected Site | Body Wall (n = 7) |
|  |  |  |  | Coelomic Fluid (n = 3) |
|  |  |  |  | Gonad (n = 3) |
|  |  |  |  | Intestine (n = 3) |
|  |  |  | Abnormal | Body Wall (n = 5) |
|  |  |  |  | Coelomic Fluid (n = 4) |
|  |  |  |  | Gonad (n = 3) |
|  |  |  |  | Intestine (n = 3) |
|  | Turks and Caicos | Diadema antillarum | Reference | Body Wall (n = 6) |
|  |  |  |  | Coelomic Fluid (n = 6) |
|  |  |  |  | Gonad (n = 5) |
|  |  |  |  | Intestine (n = 6) |
|  |  |  |  | Spines (n = 6) |
|  | United States | Diadema antillarum | Reference | Body Wall (n = 12) |
|  |  |  |  | Coelomic Fluid (n = 12) |
|  |  |  |  | Gonad (n = 12) |
|  |  |  |  | Intestine (n = 12) |
|  |  |  |  | Spine (n = 12) |
|  |  |  | Normal at Affected Site | Body Wall (n = 2) |
|  |  |  |  | Coelomic Fluid (n = 2) |
|  |  |  |  | Gonad (n = 2) |
|  |  |  |  | Intestine (n = 3) |
|  |  |  |  | Spine (n = 2) |
|  |  |  | Abnormal | Body Wall (n = 1) |
|  |  |  |  | Coelomic Fluid (n = 1) |
|  |  |  |  | Gonad (n = 1) |
|  |  |  |  | Spine (n = 1) |
|  | United States Virgin Islands | Diadema antillarum | Reference | Body Wall (n = 10) |
|  |  |  |  | Coelomic Fluid (n = 6) |
|  |  |  |  | Gonad (n = 6) |
|  |  |  |  | Intestine (n = 6) |
|  |  |  |  | Spine (n = 3) |
|  |  |  | Normal at Affected Site | Body Wall (n = 14) |
|  |  |  |  | Coelomic Fluid (n = 6) |
|  |  |  |  | Gonad (n = 6) |
|  |  |  |  | Intestine (n = 6) |
|  |  |  |  | Spines (n = 6) |
|  |  |  | Abnormal | Body Wall (n = 12) |
|  |  |  |  | Coelomic Fluid (n = 6) |
|  |  |  |  | Gonad (n = 6) |
|  |  |  |  | Intestine (n = 6) |
|  |  |  |  | Spines (n = 6) |
| **Western Indian Ocean** | Réunion Island | Echinotrix diadema | Abnormal | Coelomic Fluid (n = 13) |

**Supplementary Figure 1.** Taxonomic assessment of bacterial genera, except for *Fangia hongkongensis* which had been initially assigned as *Caedibacter taeniosporalis* group and was re-assigned through phylogenetic assessment. The graph shows taxa belonging to field collected samples that are above 5% relative abundance. The x-axis represents whether samples were grossly normal at reference site (Reference), grossly normal at affected site (Normal-AS) or DSc-Affected (Abnormal).

**Supplementary Figure 2.** Phylogenetic representation of sequences assigned to the *Caedibacter taeniosporalis* group by QIIME and the SILVA database (blue) including close relatives identified by BLASTn against the nonredundant database at NCBI. Tree is based on a 251bp overlapping portion of the 16S rRNA which was aligned with MUSCLE (Edgar, 2004). The phylogenetic reconstruction was based on Maximum Likelihood, where clustering was performed by unweighted pair-group mean average (UPGMA) following the Kimura 2-parameter model, with uniform rate nucleotide substitution method and the Nearest-Neighbor-Interchange heuristic model. Bootstrap values are based on 100 iterations of tree clustering.

**Supplementary Figure 3.** Phylogenetic representation of ASVs assigned to *Psychrobium* spp*.* by QIIME against the SILVA database (pink) including close relatives identified by BLASTn against the nonredundant database at NCBI. Tree is based on a 251bp overlapping portion of the 16S rRNA which was aligned with MUSCLE (Edgar, 2004). The phylogenetic reconstruction was based on Maximum Likelihood, where clustering was performed by unweighted pair-group mean average (UPGMA) following the Kimura 2-parameter model, with uniform rate nucleotide substitution method and the Nearest-Neighbor-Interchange heuristic model. Bootstrap values are based on 100 iterations of tree clustering.

**Supplementary Figure 4.** Core bacterial ASVs associated with (A) grossly normal (“Normal” and (B) DSc-affected (“Abnormal”) field-collected body wall specimens (“Environmental”) and challenge experiment samples of *D. antillarum* (“Challenge”). Core bacteria are defined by 0.75% relative abundance detection and 90% prevalence across the samples.
